# Supplementary material for: Identification of Freezing-Responsive microRNAs and Their Targets in Chinese Jujube by Small RNA and Degradome Sequencing
Source: Int J Mol Sci. 2026 Sep 4;27(17):7912. doi: 10.3390/ijms27177912 (PMC13566890; doi:10.3390/ijms27177912)
Supplement: Supplementary file 1 [file ijms-27-07912-s001.zip › Supplementary Material S2/novel_miR_100.pdf]

Provisional ID	:	novel_miR_100
Score total	:	1.5
Score for star read(s)	:	-1.3
Score for read counts	:	0
Score for mfe	:	1.2
Score for randfold	:	1.6
Score for cons. seed	:	
Total read count	:	55
Mature read count	:	49
Loop read count	:	6
Star read count	:	0

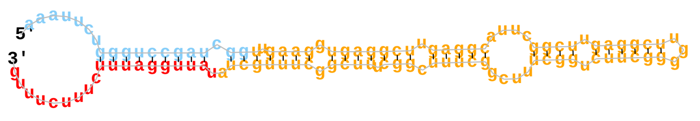

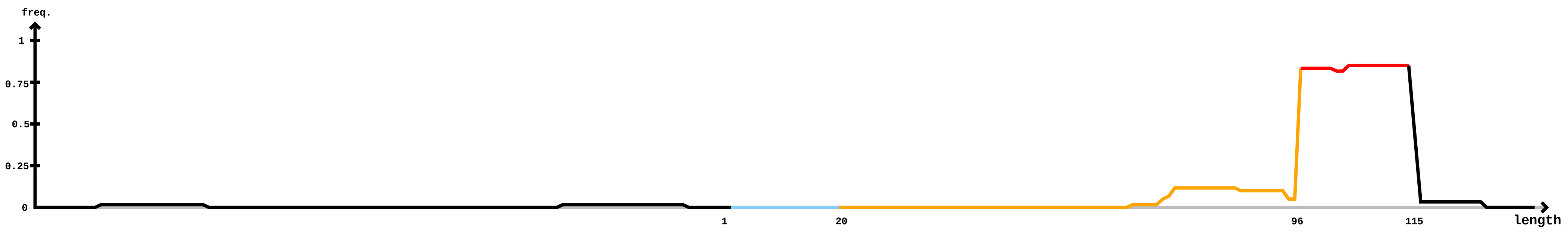

Star

Mature

```
5'- uuuacauuggauuuggauaaugggcuuuuuuguuggguaaaguuuuguaaacuggauuggaggauccaagcccuuuguuuuccgaaaaucuacaguagaaccuucgagaagucaaaauucuggguccgaucgguugaaggugaggcuugaggcauucggcuugaggcuugggggcuucuggcuuucggcuuucggcuuucggcuuugcuauauuggauuucuuucuuuguucucugguucaacguuccag -3'
```

exp: ..(((..((((((((((.(((((((((..((.((.(((..(((((...)))))..))).)).))...))))))...))).)))))..)))))..)))((((((..(((((..((((.....((((((((.(((.((((.(((((((.(((((....((((.((((((...)))))).))))....))))).))).)))).)))))))..)))))))))).....))))))))).))))))..........

read	reads	mm	sample
Aauuuggauaaugggcuu	1	1	LT-3
aaucuacaguagaaccuucga	1	0	LT-3
uuucggGuuucggcuuuc	1	1	LT-1
gcuuucggcuuucggcuuugcua	2	0	LT-3
cuuucggcuuucggcuuugcuauauugg	1	0	JCK-1
uuucggGuuucggcuuugc	3	1	LT-3
uCuuggauuucuuucuuug	1	1	LCK-1
uauuggauuucuuNcuuug	1	1	LCK-1
uauuggauuucuuucuuug	21	0	LCK-1
uauuggauuucuuucuuug	26	0	LCK-2
uucuuucuuuguucucuggguuc	2	0	LT-1
